# Supplementary material for: Signs Indicative of Central Sensitization Are Present but Not Associated with the Central Sensitization Inventory in Patients with Focal Nerve Injury
Source: J Clin Med. 2022 Feb 18;11(4):1075. doi: 10.3390/jcm11041075 (PMC8876893; doi:10.3390/jcm11041075)
Supplement: Supplementary file 1 [file jcm-11-01075-s001.zip › jcm-1583665-supplementary.pdf]

Supplementary

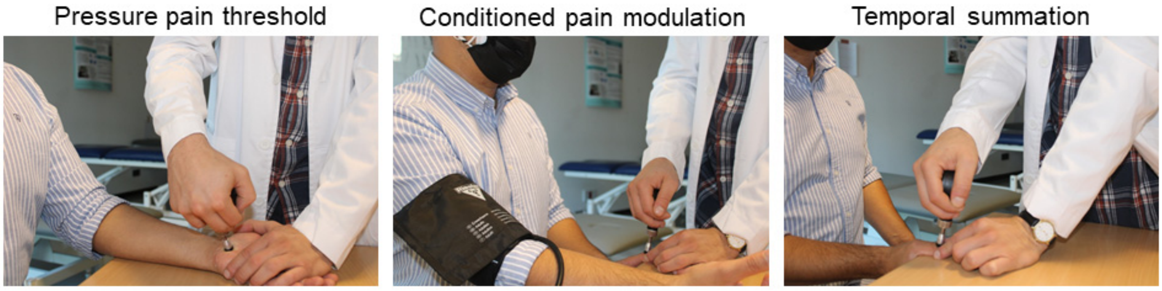

Figure S1. Three psychophysical measurements.

Table S1. Results of emotional wellbeing questionnaires.

	**Healthy (n = 30)**	**CTS (n = 30)**	***p*-Value**
BECK	3.7 [3.6]	7.9 [4.9]	0.094
STAI	27.4 [3.9]	24.3 [5.0]	0.304
TSK	20.3 [5.2]	25.9 [7.6]	0.082

Data are shown as mean [standard deviation]. *p*-values reflect Student’s *t*-tests.
